# Supporting the Implementation and Addressing the Challenges of the EU HTA Regulation

**DOI:** 10.3390/jmahp14020035

**Published:** 2026-06-08

**Authors:** Elaine Julian, Mondher Toumi, Fabrizio Gianfrate, Renato Bernardini, Stefano Capri, Mira Pavlovic-Ganascia, Oriol Solà-Morales, Antonella Cardone, Emilia Strycharz-Angrecka, Valentina Strammiello, Jean-François Bergmann, Daniel Widmer, Walter Van Dyck, Frank-Ulrich Fricke, Patrick Tilleul, Jörg Ruof

**Affiliations:** 1 Secretariat, European Access Academy (EAA), Hauensteinstr. 132, 4059 Basel, Switzerland; 2 CEReSS/UR3279—Health Services Research and Quality of Life Center, Aix-Marseille University, 13385 Marseille, France; 3 Department of Health Economics, University of Ferrara, 44121 Ferrara, Italy; 4 School of Medicine, University of Catania, 95124 Catania, Italy; 5 School of Economics and Management, Cattaneo-LIUC University, 21053 Castellanza, Italy; 6 Sabouraud Research and Treatment Centre for Scalp and Skin Diseases, St. Louis Hospital, 75010 Paris, France; 7 HiTT Foundation, 08015 Barcelona, Spain; 8 Cancer Patients Europe (CPE), 1000 Brussels, Belgium; 9 InovIntell, 30-415 Krakow, Poland; 10 European Patients’ Forum (EPF), 1040 Etterbeek, Belgium; 11 Faculty of Medicine, Université Paris Cité, 75010 Paris, France; 12 European Union of General Practitioners (UEMO), 1000 Brussels, Belgium; 13 Healthcare Management Center, Vlerick Business School, 1210 Brussels, Belgium; 14 Faculty of Business Administration, Georg Simon Ohm University of Applied Sciences Nuremberg, 90489 Nuremberg, Germany; 15 APHP and Faculty of Health (Former Member), University of Paris, 75006 Paris, France; 16 Institute for Epidemiology, Social Medicine and Health System Research, Medizinische Hochschule Hannover, 30625 Hanover, Germany

Four years after the European Regulation on Health Technology Assessment (EU HTAR) came into force and a little over a year after the implementation phase of the Regulation started, with the publication of the first Joint Clinical Assessment (JCA) report imminent, it is good timing to take a step back and revisit what has been achieved and look at what the main challenges on the way forward are.

The necessity of the regulation and the need to approach a joint European HTA assessment are undisputed. Equally, the aim of the regulation as laid out in recital 3 of the regulation is widely accepted: ‘HTA is able to contribute to the promotion of innovation, which offers the best outcomes for patients and society as a whole, and is an important tool for ensuring proper application and use of health technologies.’ [1].

The preparation phase of the regulation, which lasted until January 2025, was characterized by an enormous effort by all involved stakeholders to prepare for a successful implementation. The respective roadmap by DG Santé has been shared and updated continuously to track the key achievements and provide an up-to-date status of the implementation [2]. In addition, all other involved collaborators and stakeholders demonstrated an extensive effort to get ready for EU HTA (contributions 1–5) [3,4,5,6,7,8,9,10]. Patient advocacy groups conducted pilot Population/Intervention/Comparator/Outcome (PICO) exercises, medical societies such as the European Hematology Association tried their best to ensure expert input in the upcoming Joint Scientific Consultation (JSC) and JCA procedures, the European Medicines Agency collaborated with EU HTA on a wide variety of subjects, some—not all—EU Member States updated their national procedures to integrate JCA reports in their national processes, and, last but not least, the Health Technology Developers (HTDs) prepared their respective organizations to be ready for EU HTA and provided extensive input through various channels such as the EU HTA Stakeholder Network. The Topical Collection on EU HTA of the *Journal of Market Access and Health Policy* provides comprehensive insights into this enormous multi-stakeholder effort over the past four years. This effort is a key prerequisite to turn joint European HTA into a success.

However, many challenges prevail, and even more—some new challenges became evident during the preparation and early implementation phase for EU HTA:

- The EU HTAR covers both Medical Devices and Medicinal Products. Fundamental characteristics of those two Health Technologies are substantially different, making it very difficult to combine the future advice and assessment processes under one overall umbrella of the EU HTAR. Lifecycles of Medical Devices and Medicinal Products, the related (comparative) evidence paradigms, regulatory procedures, Member State implementation and reimbursement schemes, etc., differ considerably (contribution 6) [11]. It remains highly questionable if the same methodology guidances can be applicable to both Medical Devices and Medicinal Products [12]. Furthermore, key elements of the EU HTAR in Medical Devices share an interface with the Medical Device Regulation, causing further challenges and uncertainty [13]. The delay in implementation of the EU HTAR for Medical Devices (i.e., implementation starting one year later, in 2026) may be just the starting point for very different pathways to HTA across Medical Devices vs. Medicinal Products.
- Also, implementation of the two key activities stipulated by the EU HTAR, JSC and JCA is not appropriately balanced. An equal focus on the conduct of JSCs is required to allow HTDs to adjust their clinical development programs to the JCA needs and to meet EU HTA standards for evidence generation. However, due to lack of capacity, the JSC instrument is currently deprioritized by the Coordination Group (CG) and the Member States [14]. Consequently, the number of advice slots has been limited and only two request periods were available in 2025, and four request periods are planned for 2026 [15]. Recital 1 of the EU HTAR suggests that ‘The development of health technologies is a key driver of economic growth and innovation in the Union and is key to achieving the high level of health protection that health policies need to ensure for the benefit of all.’ [1]. The apparent limitations of early interaction opportunities within the JSCs between the CG and the HTDs are a major challenge to innovation and not in line with the intention of the regulation.
- The process that is outlined in the EU HTAR is driven by the MS. DG Santé is acting as a secretariat with the CG and the MS being in charge of conducting the JSC and JCAs. However, national market access schemes, prior approaches to HTA, methodological skill set, and, last but not least, capacities are unequally distributed across the EU Member States. Consequently, participation and involvement in the EU HTA processes are unequally distributed. A lack of Eastern European representatives in the leadership team of the CG and its subgroups was noted but has recently been addressed with the election of Mira Ludwikowska of the Polish Agency for Health Technology Assessment and Tariff System (AOTMiT) as Co-Chair of the JSC Subgroup. For EU HTA to become a success, all MS need to be involved and participate.
- Furthermore, meaningful involvement of patients and clinical experts not only on an individual level, but most importantly on an organizational level—representing consolidated expert perspectives rather than individual views—throughout the whole JSC and JCA process has to be established to ensure people centeredness and scientific excellence of the EU HTA process. Full transparency of any existing Conflict of Interest is mandatory. Nevertheless, the aim to leverage the best available external expertise to support the assessment should not be compromised.
- A vast variety of methodological guidance documents were developed and presented by the CG and its methodological subgroup [12]. This is an important step forward, as clear methodological guidance is required throughout the EU HTA process. However, key concerns have been raised regarding the evidence base of the presented methodologies (contributions 7–8). Experience from the first JSC and JCA procedures will reveal the applicability of the provided methodological guidance documents to the features of the innovative Medicinal Products in scope of the EU HTAR and their respective development programs. The bottom line remains that ‘innovation that offers best outcomes for patients and the society as a whole’ will require excellence in both the development of the Health Technologies as well as the development of applicable Assessment Methodologies [1].
- Finally, procedural challenges need to be managed and overcome. As the EU JCA largely occurs in parallel with the regulatory assessment by EMA, considerable uncertainty, e.g., regarding the PICO scoping, prevails until a very late phase of the process. This is placing a major burden both on the assessors and the affected HTDs, and a high level of agility on all sides will be required to successfully manage those challenges.

When introducing the EU HTAR, the former Commissioner for Health and Food Safety, Stella Kyriakides, emphasized the relevance of a strong ‘European Health Union’ and the need for innovative technologies to reach the patients in need as quickly and equally as possible [16]. In an increasingly competitive global environment, there is no alternative to turning this regulation into a success. However, multi-stakeholder involvement and tracking to identify shortcomings and find solutions will be key to release the full potential that this regulation brings to patients, clinicians, Member States, HTDs, and all involved stakeholders [17]. Finally, beyond all scientific, methodological, and procedural details, the JSC and JCA success of EU HTA will depend on the essential interactive skillset of all participants: the willingness to listen, to learn from each other, and the capability to make concessions in order to achieve the best outcomes for patients and society as a whole.

The EU HTAR stipulates a revision of the regulation in 2028. All involved stakeholders and collaborators are continuously gathering experience with the new process. In parallel, evidence regarding the performance of the EU HTAR has to be systematically developed to identify optimization opportunities to suggest for the revision of the regulation in 2028.

## Data Availability

No new data were created or analyzed in this study.
